# High resolution mapping of protein motions in time and space with RMSX and Flipbook

**DOI:** 10.1038/s41598-026-39869-7

**Published:** 2026-02-20

**Authors:** Finn Beruldsen, Martiela Vaz de Freitas, Dinler A. Antunes

**Affiliations:** Center for Nuclear Receptors and Cell Signaling, Department of Biology and Biochemistry, University of Houston, Science and Engineering Research Center, 3517 Cullen Blvd bldg 545, Houston, TX 77004 USA

**Keywords:** Molecular dynamics (MD), RMSD, RMSF, Time-resolved fluctuation, Trajectory visualization, Biochemistry, Biophysics, Computational biology and bioinformatics, Mathematics and computing, Physics

## Abstract

Demonstrating when and where proteins undergo conformational rearrangement remains challenging in molecular dynamics (MD) analysis, particularly for transient and localized motions. We introduce **RMSX**, a time-series extension of root-mean-square fluctuation (RMSF), and **Flipbook**, a general-purpose method for mapping simulation metrics onto structural, atomistic snapshots. Unlike traditional RMSF values, RMSX is performed over partitions of the original simulation – not its entirety. This simple extension allows RMSX to pinpoint not only the degree of fluctuation but also isolate when a significant motion takes place for any amino acid residue. Flipbook provides an engine for transforming time-series, residue-level data into salient, colored 3D structures. Flipbook can take in RMSX values, or other user-provided values, to see how they vary with time, coloring and scaling the amino acids according to those values. We demonstrate these tools’ utility on unbiased and steered MD simulations of ubiquitin, HIV-1 protease, and the bacterial adhesion protein SdrG. RMSX and Flipbook together form a streamlined, open-source suite for quantitative, high-resolution interrogation of biomolecular dynamics. RMSX and Flipbook are freely available at https://github.com/AntunesLab/rmsx.

## Introduction

Understanding the precise timing and location of conformational shifts in proteins is critical for investigating mechanisms such as allostery, catch-bond formation, and mechanotransduction^1,2^. Molecular dynamics (MD) simulations generate detailed atomic trajectories, but extracting precise temporal and spatial information, specifically when and where individual residues undergo significant motion, remains challenging. These limitations impact both research discovery and scientific communication of findings related to local and transient conformational changes, particularly in printed media.

Common metrics such as Root Mean Square Deviation (RMSD) and Root Mean Square Fluctuation (RMSF) quantify global structural drift and average per residue mobility, respectively, but cannot simultaneously describe both *when* (during a simulation) and *where* (what part of the protein) structural changes occur. Alternatively, unsupervised approaches such as principal component analysis (PCA) and dynamic network models extract dominant motion modes or correlated residue networks but sacrifice interpretability or simplify motion throughout the simulation^3–5^.

Here, we introduce two open source methods, RMSX and Flipbook, that together fill this gap. RMSX partitions an MD trajectory into consecutive time slices (or windows), computes per-residue RMSF within each window, and assembles the results into a heatmap that simultaneously displays spatial and temporal variation. Flipbook projects any per-snapshot scalar (e.g., RMSX values, interatomic distances, hydrogen-bond occupancy) onto the B-factor field of the structure files (PDB format) and arranges snapshots in sequence, creating an intuitive ‘flipbook’, a sequential illustration of conformational changes (see full workflow in Supp. Fig. 1).

**Fig. 1:**
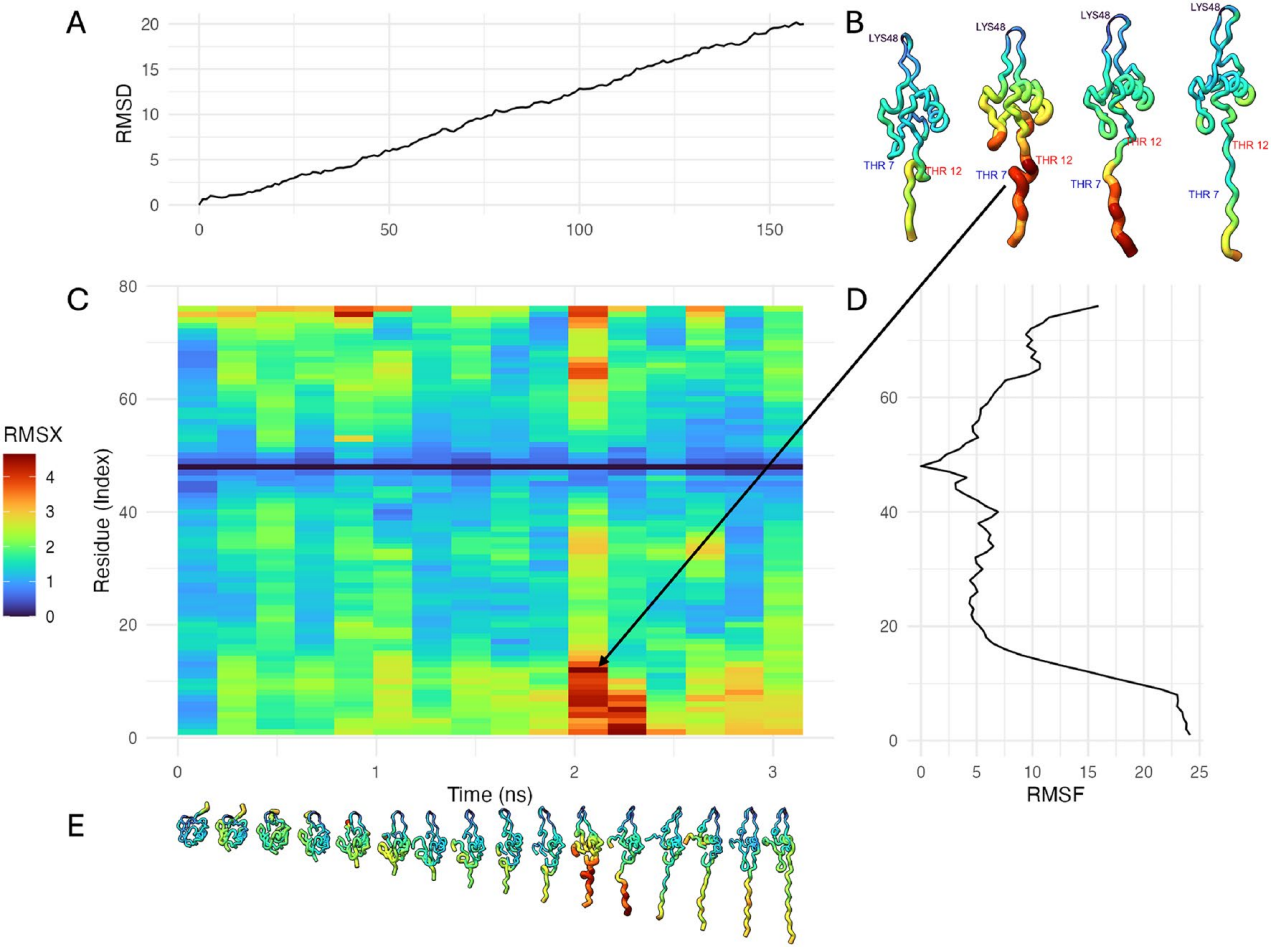
Ubiquitin protein under steered molecular dynamics (SMD), visualized with RMSX and Flipbook. (**A**) Root-mean-square deviation (RMSD) over time, showing global structural displacement in angstroms (Å) during a 3.15 ns SMD trajectory. (**B**) Representative Flipbook renderings of the structure at selected timepoints, with color and tube thickness scaled by RMSX values. Regions of higher mobility (THR7 and THR12) are highlighted in red, while the anchored rigid region (LYS48) remains dark blue across time. (**C**) RMSX heatmap displaying per-residue displacement (Å) across time. Time is plotted on the x-axis (in nanoseconds), and residue index on the y-axis. Warmer colors indicate greater positional fluctuations. (**D**) Root-mean-square fluctuation (RMSF) profile across all residues, with displacement (Å) on the x-axis and residue index on the y-axis. (**E**) Full Flipbook panel illustrating continuous motion along the trajectory, emphasizing both when and where fluctuations occur.

We illustrate these workflows on (i) steered molecular dynamics (SMD) of ubiquitin denaturation, (ii) unbiased MD simulations of HIV-1 protease, and (iii) SMD of the bacterial adhesin SdrG. Our implementation is built on MDAnalysis^6^ and integrates seamlessly with major MD visualization suites, including ChimeraX^7^ and VMD^8^, producing publication-quality visualizations with minimal setup. RMSX and Flipbook provide a unified pipeline for high-resolution, quantitative interrogation of biomolecular dynamics, enabling researchers to identify and communicate complex conformational events in print media.

## Design and implementation

### RMSX: time-resolved fluctuation analysis

RMSX is a Python-based tool that generates time-resolved, per-residue fluctuation heatmaps (Fig. 1) directly within Jupyter notebooks^9^, providing an interactive and reproducible environment for MD analysis. It leverages the MDAnalysis library^6^ for efficient trajectory parsing and manipulation, and produces publication-ready visualizations using R’s ggplot2^10^, and UCSF ChimeraX^7^ or VMD^8^, for high-quality 3D structural renderings. The use of MDAnalysis^6^ ensures compatibility with all major MD engines, including GROMACS^11^, NAMD^12^, LAMMPS^13^, CHARMM^14^, and Amber^15^. By combining Python-based data handling with external graph generation and visualization tools, RMSX supports interactive exploration of residue-level dynamics and the generation of high-quality figures.

#### Data inputs and outputs

RMSX requires only a structure file and a trajectory file. PDB files for each chain are generated and sorted into directories by chain. The all_chain_rmsx() function performs RMSX analysis across all chains in a complex, generating outputs for each chain individually as well as for the full complex. RMSX values for each residue are stored in the B-factor column of each PDB and exported as a CSV table. In summary, for each chain, the following outputs are produced:

- An RMSX heatmap (PNG).
- A chain_rmsx.csv file of per-residue, per-slice values.
- PDB snapshots with RMSX values stored in the B-factor column.

The scripts automatically save a PNG plot for each chain analyzed; if Flipbook is used, a high-quality PNG of the snapshots from UCSF ChimeraX are produced with the option to use VMD to produce photorealistic molecular visualizations with features like ambient occlusion lighting and depth cueing^16^. Users can adjust the snapshots’ orientation or visualization via the UCSF ChimeraX GUI or customized VMD shortcuts, and resave transparent-background images by rerunning the save command.

The run_rmsx() function performs the same process for a single specified chain at a time, prompting the user with a list of available chains (Supp. Code Snippet 1).

#### Trajectory slicing

RMSX reads a trajectory (.xtc, .dcd, etc.) and divides it into either a user-specified number of slices or a fixed number of frames per slice. If the trajectory length is not evenly divisible, excess frames at the end are dropped. RMSX reports both the total frames and equivalent simulation time (ns), showing exactly what was analyzed. You can also supply start_frame and end_frame to exclude equilibration or focus on a particular time segment.

#### Visualization options

Users may enable interpolation between slices and choose from colorblind-friendly viridis palettes (e.g., “magma,” “plasma”) that are consistently applied across the heatmap and UCSF ChimeraX or VMD renderings.

**Fig. 2:**
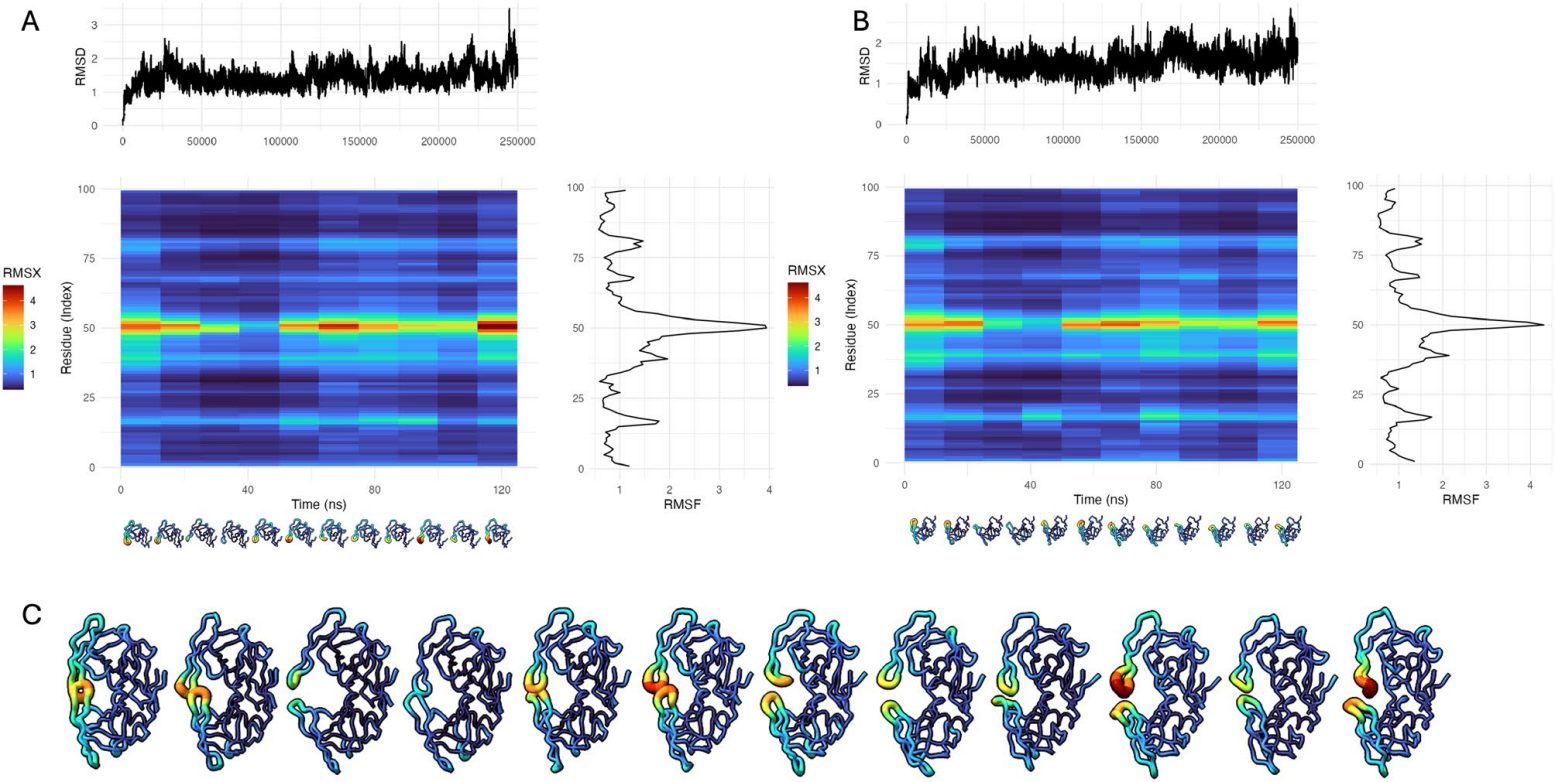
Protease 125 ns simulation: Highlighting regions of higher and lower molecular fluctuations. (**A**) RMSX analysis of Chain A, shown as a triple plot. Top: RMSD trajectory (y-axis in Å) plotted over simulation time (x-axis in ns). Middle: Heatmap of RMSX values per residue across time (in ns), where warmer colors indicate greater fluctuations. Right: RMSF profile summarizing the average displacement per residue. Structural snapshots below the heatmap show per-slice RMSX-mapped conformations, with fluctuation magnitude encoded by tube thickness and color done using Flipbook. (**B**) Equivalent analysis for Chain B, including RMSD, RMSX heatmap, RMSF profile, and per-slice structural renderings. (**C**) Flipbook visualization of the full protease dimer (Chains A and B), with both chains shown together at matched timepoints. Regions of high flexibility–consistently observed in both chains–are clearly visible through localized red/yellow coloring, while structurally rigid areas remain blue.

**Fig. 3:**
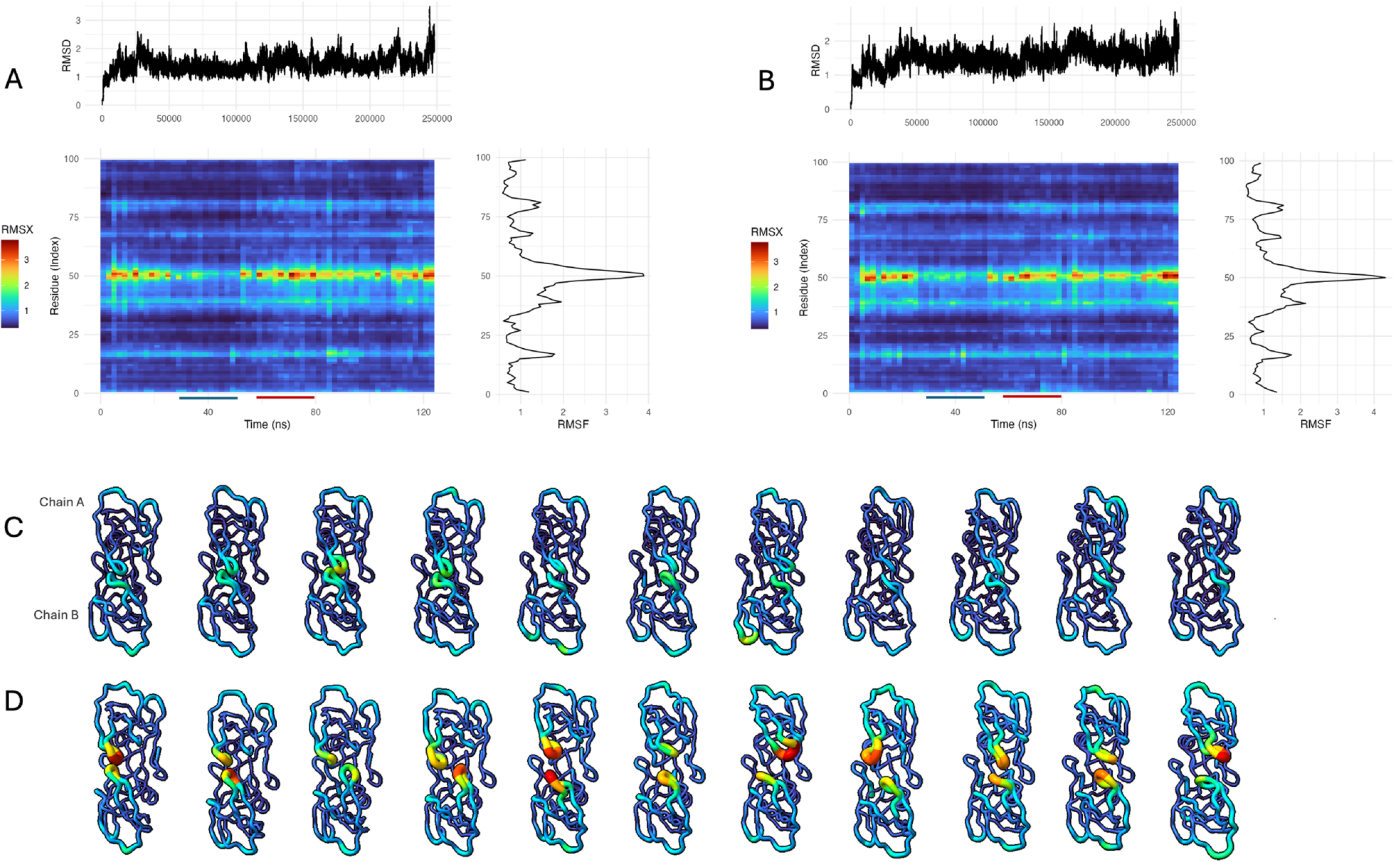
RMSX fluctuations reveal distinct dynamic states in protease dimer: open vs. closed. (**A**) Triple plot for RMSX analysis of Chain A over a 125 ns GROMACS simulation. The top panel shows RMSD vs. time (ps), the middle heatmap presents per-residue RMSX values (in Å) over time (ns), and the right panel displays RMSF vs. residue index. Regions of high residue motion (red) and low fluctuation (blue) are underlined for clarity. (**B**) Triple plot for Chain B, as in A, highlighting similar dynamic regions with both chains showing elevated motion around residue $\tilde{5}0$. (**C**) Flipbook visualization of a region with consistently low RMSX values, showing minimal structural fluctuation across timepoints. Chains A and B are shown together in each frame. (**D**) Flipbook of a region with high RMSX values, corresponding to flexible or transiently mobile segments of the protease. The same chains are displayed as in Fig. 2C, but rotated  $90^{\circ }$ around the y-axis for clarity. Color and thickness reflect RMSX magnitude (in Å), as encoded in the heatmap scale.

#### Contextual RMSD/RMSF plots

Optionally, RMSX can generate flanking RMSD and RMSF plots alongside the heatmap. RMSD summarizes global structural drift, while RMSF provides average per-residue mobility; together, they give context to the time-resolved heatmap.

### Flipbook: time-resolved molecular dynamics visualization

Flipbook is a lightweight, customizable tool designed for visualizing time-resolved molecular dynamics by creating sequential, per-snapshot representations of structural metrics within UCSF ChimeraX or VMD.

#### Core functionality

Flipbook reads structural metrics (such as RMSX computed over defined time slices, interatomic distances, or hydrogen-bond occupancy) encoded in the B-factor column of PDB files. It then automatically loads, aligns, and arranges these snapshots in sequence, creating a spatial composite–effectively a “flipbook” view of conformational dynamics.

The core principle is to map the time-resolved metric onto the structure’s visualization:

- For each snapshot, residue color and size are scaled to the corresponding metric value, immediately highlighting the spatial and temporal evolution of fluctuations or other dynamic features (case examples in Supp. Fig. 2-3).
- Consistent color, value, and scaling across snapshots are essential for creating representative, publication-ready composite images.

#### Implementation and viewer support

Because no single molecular visualization suite natively supports all required features, Flipbook coordinates ChimeraX and VMD through a combination of Python and viewer-specific scripting.

*UCSF ChimeraX*: provided support for most required features, simplifying the alignment and arrangement process. However, to ensure consistency between the heatmaps and the Flipbook visualization, we implemented custom color scales. Advanced users can further customize visualizations by providing ChimeraX scripts, for example, to highlight specific chains or freeze parts of the structure while mapping dynamic changes onto others.

*VMD (Visual Molecular Dynamics)*: We wrote a custom visualization method using TCL scripting to appropriately scale the size of each residue based on its metric. We worked closely with the developers of VMD to bring support for Flipbooks within their ecosystem. The inclusion of VMD allows users a far greater degree of customizability, access to advanced raytracing options through Tachyon^16^, and lower-level control of the molecular visualizations^8^. We developed a set of commands and shortcuts to allow users to easily control the spacing, alignment, and simultaneous rotation of the snapshots, compensating for VMD’s lack of support for multiple protein-specific pivot points.

With this custom scripting, Flipbook outputs a high-resolution, publication-ready composite image of the full trajectory from either viewer.

## Case studies and benchmarks

### Dynamic profiling of ubiquitin under SMD

We applied RMSX and Flipbook to a Steered Molecular Dynamics (SMD) trajectory of ubiquitin to observe and visualize the spring-like time-resolved unfolding of the protein under application of external force.

#### Simulation setup

We analyzed a 3.15 ns SMD trajectory of ubiquitin (PDB:1UBQ) from the “Case Study: Ubiquitin” dataset^17,18^. As reported by the authors, the simulation was run in NAMD version 2.14, with PME electrostatics and periodic boundary conditions. The Lys48 residue was held fixed while Met1 was steered at 0.05 Å/ps using a 208.4 pN/Å spring constant. Frames were recorded every 10 ps, and waters were stripped for analysis.

#### RMSX and Flipbook analysis

We processed the first 150 frames ($\sim$1.5 ns) using a sliding window of 10 frames in run_rmsx_flipbook() (Supp. Code Snippet 2), generating an RMSX heatmap and per-slice data. Flipbook snapshots were exported with the calculated RMSX values placed in the B-factor column and rendered using customized scripts for either UCSF ChimeraX or VMD, where residue size and color were scaled proportionally to the RMSX value (“Worms” style). To ensure consistent color mapping between heatmaps and flipbooks, we ported the viridis palettes from R (magma, plasma, inferno, cividis, mako, rocket, turbo) into ChimeraX^19^.

Our analysis shows the time-dependent nature of the forced unfolding (Fig. 1). Fluctuation is concentrated near the N- and C-termini, peaking around frame 103 ($\sim$1.05 ns) with a maximum RMSX of $\sim$4.6 Å, while the fixed anchor residue Lys48 remains immobile (Fig. 1). The Flipbook visualization highlights this spring-like unfolding, visually showing residues like Thr7 swinging away from Thr12 at peak RMSX.

### Sampling transient shifts around the semi-open conformation of protease

The HIV-1 protease is a homodimeric enzyme essential for viral maturation and a key target in antiretroviral therapy. Its catalytic activity relies on the dynamic opening and closing of two flexible flaps that control substrate and inhibitor access to the active site. As shown by^20^, structural changes at specific residues can dramatically impact protease dynamics. We explored the dynamic motions of the protease flaps to test if RMSX would be able to show the transition between semi-open and closed conformations of the wild-type protein. This activity relies on the dynamic opening and closing of two flexible flaps that control substrate and inhibitor access to the active site^20^.

#### Simulation setup

We analyzed a 125 ns GROMACS trajectory of the wild-type HIV-1 subtype B protease (PDB: 1OHR), which was run with the charmm36 force field, tip3p water model, and final ion concentration of 0.15 mol/L. The system was energy minimized and equilibrated following a previously described protocol^21,22^. Snapshots were recorded every 25,000 frames (250,000 frames total).

#### RMSX an Flipbook analysis

Initial analysis using run_rmsx() with 10 equal slices (25,000 frames each) concentrated the fluctuation in the “tip of the flap” (around position 50 of each chain), a key indicator of enzyme dynamics (Fig. 2C). To investigate the more granular details of areas of association/dissociation between the flap tips, we refined our analysis with a smaller slice size of 4,000 frames (yielding 62 slices, Fig. 3A,B). This higher temporal resolution allowed us to distinguish between two key periods. First, a *Calm Period* (Slices 16-26; frames 64,000-10,4000) which shows minimal RMSX at residue position 50, indicating a stable closed state (Fig. 3C). Then, a *Dynamic Period* (Slices 35-45; frames 140,000-180,000) which shows elevated RMSX values around the active site, reflecting transient flap opening events (Fig. 3C,D).

This analysis pinpoints the precise timing of flap tip motions for this dimeric protein, which can be affected by specific protease mutations^20^. In turn, the dynamic behavior of the flaps for different protease variants may impact substrate access to the catalytic site of the enzyme, or the rates of inhibitor binding/unbinding events^20^. This highlights the utility of the RMSX and Flipbook for data exploration and the identification of key positions and time periods within MD simulations.

**Fig. 4:**
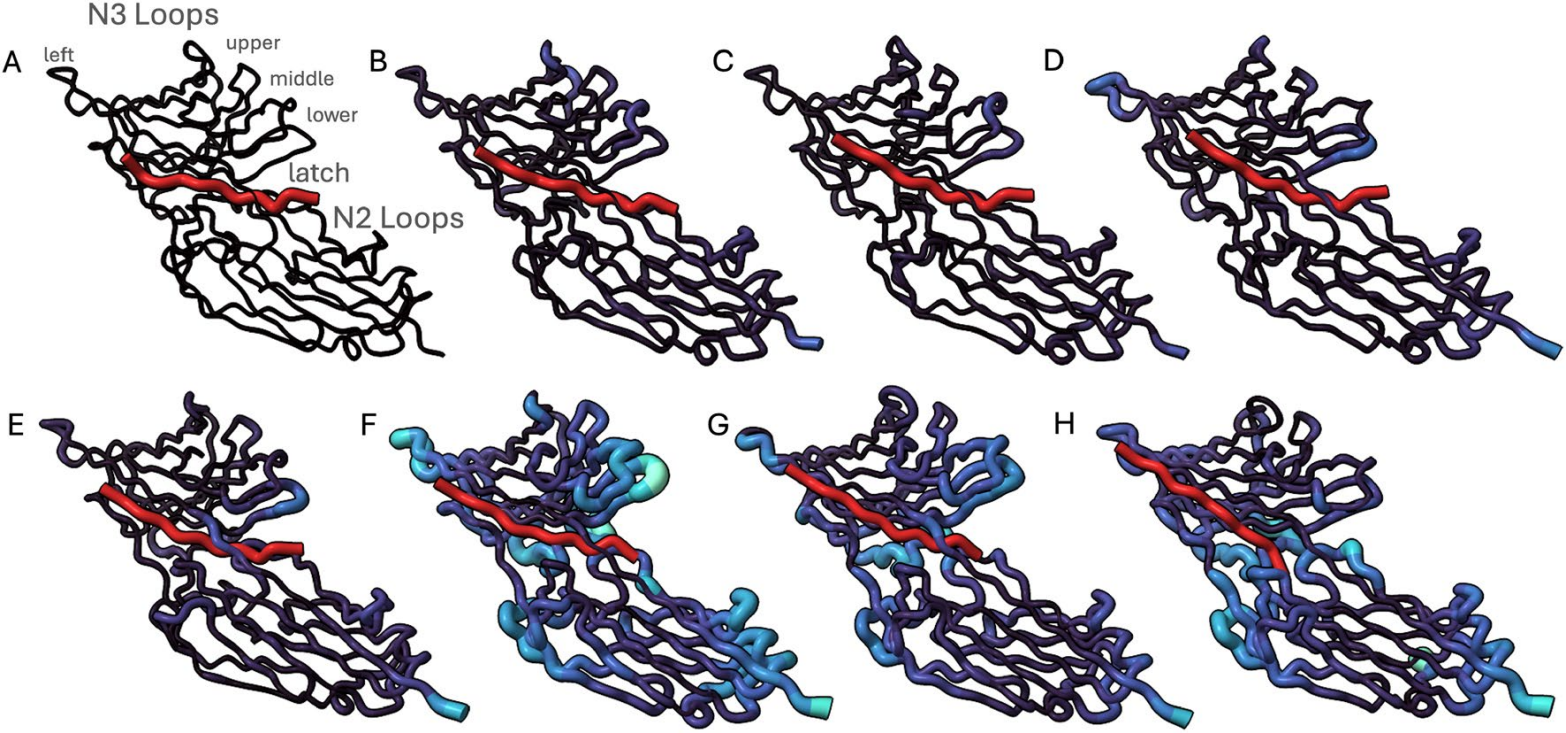
A Flipbook of molecular distances of SdrG from a high-force dissociation simulation. Peptide is colored in red, for the SdrG complex, both color and size of each residue are proportional to their shift from their starting position. (**A**) First frame of the simulation (zero shift reference). (**B**) Movement of the upper and middle loops of the N3 complex. (**C**) Shift in lower and middle N3 loops, relaxation of upper loops. (**D**) shift of lower and left N3 loops. (**E**) Relaxation of left N3 loop, shift of lower N3 loop, shift of latch portion in contact with peptide. (**F**) confirmational shifts throughout the exterior portion of the (**G**) relaxation of complex, still notable shift in left, middle, and lower N3 loops and N2 loops beneath the latch. (**H**) N2, particularly below the latch, remains shifted out of place, N3 loops further relax towards their starting position.

### Visualizing SdrG shifts under a high-force regime

SdrG is a fibrinogen-binding adhesin from *Staphylococcus epidermidis* that binds to the $\beta$-chain of human fibrinogen with extreme mechanical resilience. Structural and single-molecule studies have shown that its “dock, lock, and latch” mechanism forms a non-covalent complex capable of withstanding forces exceeding 2 nN, making it one of the strongest known receptor–ligand interactions^23–25^. This exceptional stability arises from a catch-bond effect in which applied force prolongs bond lifetimes^26^, making SdrG a biologically relevant system for testing time-resolved visualization methods.

#### Simulation setup

We analyzed SMD trajectories from Milles et al. (2018)^24^ in which the SdrG–fibrinogen complex was pulled along the $-z$ axis at 0.025 Å $\textrm{ps}^{-1}$ using a harmonic spring constant of $0.15\,\mathrm {kcal\,mol^{-1}}$ Å$^{-2}$.

#### RMSX, Shift Maps and Flipbook analysis

To provide a comprehensive view of the mechanical response, we combine three analytical and visualization tools: RMSX, Shift Maps, and Flipbook.

*RMSX and Flipbook visualization*: First, we applied Flipbook to these trajectories, which uses the time-resolved RMSX values to reveal the stepwise loop movements that underlie the mechanostability of SdrG.

*Per-residue Shift Maps visualization*: Then, to complement the RMSX fluctuation analysis, we incorporate per-residue “shift maps” (a.k.a. trajectory maps) following Kozic et al.^27^. A shift map computes the Euclidean displacement of each residue from a chosen reference frame–typically the initial structure–thereby capturing the cumulative drift over the simulation. This drift can also be visualized over the 3D stapshots of the protein using Flipbook.

The force-induced tightening of the N3 latch loop in SdrG is evident as a pronounced high-magnitude red patch in the shift map. The corresponding Flipbook frames reveal the gradual ordering and displacement of the complementarity-determining region over time. This combined approach, as seen sequentially in Fig. 4, therefore provides a quantitative displacement profile (Shift Map) to pinpoint which loops undergo the largest net movement. It also provides a dynamic structural illustration (Flipbook) to observe exactly when those displacements and local fluctuations occur, providing a full narrative of the molecular events underlying mechanostability.

### Benchmarking RMSX against established metrics

RMSX is, by construction, a time-resolved extension of RMSF: within each time window, it computes a standard RMSF per residue, and the RMSX heatmap is simply the concatenation of these window-wise RMSF profiles. Because of this, RMSX should behave consistently with conventional RMSF and RMSD while adding temporal resolution. At the same time, template-based metrics such as shift (trajectory) maps and local distance difference test (LDDT) scores capture complementary aspects of the dynamics. In this section, we benchmark RMSX against these established quantities and outline when each metric is most informative.

### Consistency with conventional RMSF and RMSD

As a first sanity check, we asked whether RMSX reproduces the same residue-wise mobility patterns as a conventional, trajectory-wide RMSF calculation. For each system (ubiquitin, HIV-1 protease, SdrG), we computed: (i) standard RMSF over the full trajectory and (ii) the time-averaged RMSX per residue (i.e., the mean across all slices in the RMSX matrix).

Across all three systems, the mean RMSX and classical RMSF profiles were strongly correlated. For the ubiquitin SMD trajectory, the Pearson correlation between per-residue RMSF and mean RMSX was $r = 0.871$ ($R^2 = 0.759$). For the HIV-1 protease, the corresponding correlations in the 12-slice analysis were $r = 0.996$ ($R^2 = 0.992$) for chain A and $r = 0.992$ ($R^2 = 0.984$) for chain B. A finer-grained analysis using 4000-frame windows gave similarly high correlations: $r = 0.990$ ($R^2 = 0.981$) for chain A and $r = 0.982$ ($R^2 = 0.965$) for chain B. For the SdrG high-force SMD trajectory (Supp. Fig. 6:B), the correlation between RMSF and mean RMSX was somewhat lower but still substantial, $r = 0.783$ ($R^2 = 0.614$). In all cases, peaks corresponding to flexible loops and termini coincide in both traces (Supp. Fig. 6:A,B), confirming that RMSX faithfully recovers the underlying fluctuation profile when averaged over time.

At the global level, RMSX-derived summaries were also broadly consistent with RMSD, although the strength of this relationship depended on the system and on the nature of the motion. For each system, we compared the time series of the mean RMSX per slice (averaged over residues) to the backbone RMSD relative to the starting structure. In ubiquitin, the correlation between mean RMSX and mean RMSD was modest ($r = 0.245$, $R^2 = 0.060$). For the HIV-1 protease, correlations ranged from $r = 0.312$ ($R^2 = 0.097$) for chain A to $r = -0.168$ ($R^2 = 0.028$) for chain B in the 12-slice analysis, and from $r = 0.483$ ($R^2 = 0.233$) for chain A to $r = 0.257$ ($R^2 = 0.066$) for chain B in the 4,000-frame analysis. Despite these low-to-moderate correlation coefficients, the mean RMSX and RMSD curves still highlight the same major opening/closing events in the triple plots (Supp. Fig. 6).

RMSD and RMSX are related measures of structural motion, but the strength of their relationship depends on whether the underlying dynamics are collective or local. RMSD is a template-based measure of net structural drift, whereas RMSX reports *within-window* positional fluctuations. The two tend to correlate when the dominant motion is collective, such as domain motion or steady, directed displacement during steered simulations. In these cases, the same underlying structural change produces both increased global drift (RMSD) and elevated within-window fluctuations (mean RMSX), causing the two measures to rise over the same time periods. Conversely, they can be weakly correlated or even decoupled when the trajectory is dominated by local fluctuations that do not accumulate into net drift (high RMSX with relatively stable RMSD), or when drift occurs on timescales that are slow relative to the chosen window (RMSD increases while within-window RMSX remains comparatively small).

In contrast, for the SdrG high-force pulling simulation, where the dominant motion is a monotonic forced dissociation, mean RMSX and RMSD were tightly coupled ($r = 0.879$, $R^2 = 0.772$). Taken together, these benchmarks show that: (i) RMSX is numerically consistent with classical RMSF at the residue level across all systems, and (ii) RMSD should be interpreted as global context for RMSX rather than as a strict quantitative proxy, because the RMSD–RMSX relationship depends on whether motion is dominated by collective rearrangements versus local fluctuations.

### Effect of window size and the window_check heuristic

Because RMSX is window-based, its behavior depends on the choice of window length, which governs the trade-off between temporal resolution and variance. Short windows increase temporal resolution but raise variance; long windows smooth the signal but may wash out short-lived events. To quantify this trade-off, we implemented a window_check routine for comparing multiple window sizes of RMSX-derived summaries to RMSD. For each system, we evaluated window sizes ranging from 4 to 128 slices (Supp. Fig.6). Very long windows (few slices) yielded smooth mean RMSX curves and could give relatively high correlation with RMSD (e.g. $R^2 \approx 0.88$), but visual inspection showed that they merged distinct calm and dynamic periods. Very short windows (many slices) recovered more events but produced noisier mean RMSX traces and lower correlation with RMSD (e.g. $R^2 \approx 0.46$).

Comparing the mean RMSD with mean RMSX provides a useful heuristic for tuning the visualization. While they are imperfectly correlated, trying multiple window sizes and choosing the one that best matches RMSD can make sure the most significant motions of a protein are captured. Based on these results, we recommend selecting RMSX windows on the order of one to two orders of magnitude shorter than the dominant timescale of motion inferred from RMSD, and using window_check to visually verify that: (i) the mean RMSX curve is not dominated by noise, and (ii) major RMSD transitions are still reflected in the RMSX signal. The ideal number of frames per window depends on the system flexibility and the number of frames per picosecond. Removing the equalization frames and finding a balanced window size improves the quality of the visualizations and we have provided the options start_frame window_check to explore different window sizes and compare them with RMSF and RMSD. We have found intermediate window sizes of 8-16 slices are effective for the most common types of simulations researchers run: small to moderately sized proteins with simulations on the order of 1-100 ns. For longer simulations, we recommend trying a greater number of slices with a multi-level approach like the one seen in Fig3 using Flipbook to compare time periods of interest.

### Complementarity with shift maps and lDDT

We next compared RMSX against two template-based metrics that are naturally expressed as residue-by-time matrices: shift maps (trajectory maps)^27^ and per-residue lDDT time series^28^. All three metrics can now be computed and visualized within the same pipeline, using identical heatmap and Flipbook outputs.

#### The contrast between drift (Shift Maps) and fluctuation (RMSX)

Shift maps quantify, for each residue and frame, the Euclidean displacement from a chosen reference conformation. Applied to SdrG, shift maps highlight loops that undergo large net displacements under force, particularly in the N2/N3 regions (Fig. 4D).

**Fig. 5:**
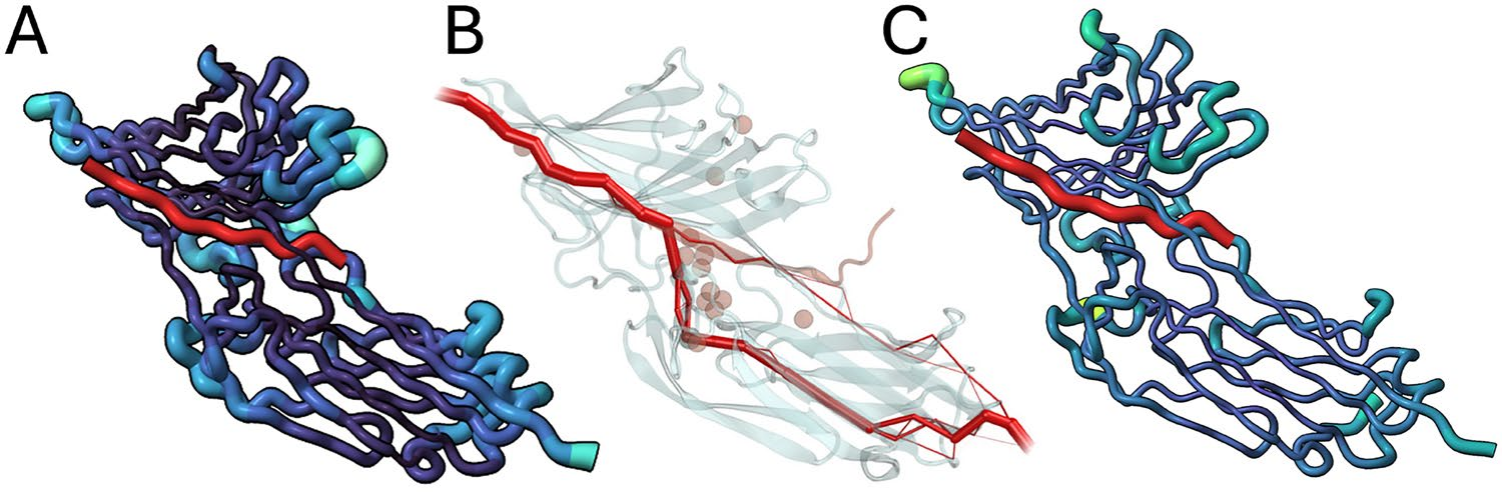
Comparison of more stable regions during large-scale conformational shifts. (**A**) from 4F, shift map of SdrG. (**B**) Dynamic network analysis of SdrG (adapted from Melo et al.^2^). (**C**) RMSX over the same time slice.

In contrast, RMSX divides the trajectory into time windows, computes per-residue RMSF within each slice, and assembles these into a heatmap that simultaneously encodes “when” and “where” fluctuations occur. RMSX reports within-window fluctuations and is insensitive to monotonic drift. For SdrG, the N2 loops accumulate substantial shift (Fig. 5A), but their RMSX remains low (Fig. 5C).

This distinction is also evident in HIV-1 protease: flap residues that repeatedly open and close exhibit strong RMSX hotspots even when their net displacement from the reference is moderate. By overlaying these metrics in our Flipbook pipeline, we obtain a unified view of cumulative drift and localized, time-resolved flexibility. Figure 5A shows the shift map for the frame exhibiting maximum global drift, while Fig. 5C shows the corresponding RMSX values–highlighting residues that remain rigid despite overall displacement.

#### The role of lDDT

Per-residue lDDT^28^ captures local neighborhood distortions by quantifying how well short-range interatomic distances are preserved relative to a reference structure. Because lDDT is insensitive to rigid-body motion, it highlights small-scale structural rearrangements such as loop repacking or side-chain reorganization. In our benchmarks, lDDT was less sensitive to motions where local atomic neighborhoods remain preserved–for example, the largely rigid-body opening and closing of the protease flaps (Fig. 3)–where trajectory maps and especially RMSX detected the transition clearly. In contrast, lDDT highlighted changes in the hinge region of protease, where local rearrangements accompany flap motion (Supp. Fig. 7).

#### Synthesis of metrics

These comparisons support a simple division of labor among the three metrics: RMSX quantifies how dynamically each residue behaves over time; shift maps report how far each residue drifts from a chosen reference and are ideal for identifying entry into or exit from specific conformations; and lDDT detects local neighborhood distortions while remaining robust to domain-level motion. By providing unified implementations of all three, together with consistent heatmap and Flipbook visualizations, our pipeline enables users to select the metric–or combination of metrics–best aligned with their scientific question.

## Conclusion

In summary, RMSX merges the strengths of RMSD and RMSF by providing time-sliced, per-residue fluctuations in a single heatmap that reveals both when and where structural changes occur. Flipbook maps these values onto 3D structures, creating an intuitive visual “flipbook” of the protein’s motion and enhancing the interpretability of time-resolved dynamics. Together, RMSX and Flipbook offer a holistic, publication-ready approach for visualizing MD simulations. Our tools, tutorials, and documentation are freely available at GitHub (https://github.com/AntunesLab/rmsx).

Our tool performed effectively across three different systems, encompassing diverse protein sizes, numbers of chains, and multiple MD platforms. It operates efficiently, producing results in seconds for small proteins and minutes for larger simulations, and clearly highlights regions of structural motion over time. Designed with accessibility in mind, it enables researchers with a range of technical backgrounds to generate publication-quality visual summaries of protein dynamics.

RMSX values can be directly mapped onto structural snapshots using Flipbook–a strategy that also generalizes to shift maps and lDDT. While shift maps detect cumulative displacement and lDDT captures local structural rearrangements, RMSX pinpoints the timing and localization of transient fluctuations. Very slow drifts that span longer than a single window may be attenuated in RMSX plots, whereas shift maps or lDDT remain sensitive to these types of motion. These methods are therefore complementary: shift maps and lDDT track cumulative or local distortions relative to a baseline, while RMSX excels at resolving short-lived or intermittent events. Used together, they provide a powerful and accessible framework for understanding protein motions and the temporal progression of conformational changes in MD simulations.

## Supplementary Information

Supplementary Information.

## Data Availability

The code and relevant files are publicly available on GitHub: https://github.com/AntunesLab/rmsx.
