# Supplementary material for: High resolution mapping of protein motions in time and space with RMSX and Flipbook
Source: Sci Rep. 2026 Feb 20;16:10035. doi: 10.1038/s41598-026-39869-7 (PMC13022101; doi:10.1038/s41598-026-39869-7)
Supplement: Supplementary file 1 — Supplementary Information. [file 41598_2026_39869_MOESM1_ESM.pdf]

Supplementary Information

Supplementary Methods

Running RMSX: Full Python Example

The examples on Supplemental Codes 1 and 2 demonstrate the complete invocation of the `run_rmsx` function from our Python API. This will:

1. Slice a trajectory into 9 equal windows,
2. Compute per-residue RMSF in each slice,
3. Apply a log-transform to the RMSX values,
4. Generate a heatmap (using the "mako" palette) and export PDB snapshots with RMSX in the B-factor column.

Argument highlights

- `num_slices` vs. `slice_size`: specify either the # of windows or the exact frames/window.
- `interpolate=True`: smooths abrupt transitions when plotting.
- `triple=True`: adds flanking RMSD and RMSF plots for context.
- `palette`: must match one of our ported viridis palettes (e.g. "mako," "magma," "plasma").
- `log_transform=True`: apply a $\log_{10}$ transform to RMSX before plotting—useful for high-dynamic-range datasets.

After this call completes, you will find in `output_dir`:

- `chain_rmsx.csv`
- `heatmap.png`: publication-quality RMSX heatmap,
- `snapshots/*.pdb`: one PDB per slice with RMSX in the B-factor column.

Choosing Window Size, Our Recommendations

Best practices: first ensure that your equilibration phase isn't included in the simulation. We recommend 5–10 ns windows (10 000–25 000 frames) to capture both transient and sustained motions. Smaller windows highlight brief events but increase noise; larger windows smooth fluctuations but may obscure rapid transitions.

Custom Legend Labels

In `create_r_plot.R`, we added a `fill_label` argument so users can override the default "RMSX" or "Log-Scaled RMSX" legend. If unset, defaults are retained. Our Python wrapper passes "Shift map Å" for shift-map analyses.

Supplemental Code Snippet 1: Example invocation of `run_rmsx()`.

```
from rmsx import run_rmsx

# File paths
pdb_file   = "/path/to/structure.pdb"
dcd_file   = "/path/to/trajectory.dcd"
output_dir = "/path/to/results/rmsx_run"

# Run RMSX
run_rmsx(
    topology_file      = pdb_file,          # your PDB/PSF
    trajectory_file    = dcd_file,          # your DCD/XTC, etc.
    output_dir         = output_dir,        # where to save results
    num_slices         = 9,                 # split trajectory into 9 windows
    slice_size         = None,              # alternative: fixed frames/window
    rscript_executable = "Rscript",          # path to your Rscript (Advanced feature
        )
    verbose            = True,               # print progress
    interpolate        = True,               # smooth between slices
    triple             = False,              # add flanking RMSD/RMSF plots
    chain_sele         = None,               # e.g. "chain A", or None for all
    overwrite          = False,              # replace existing outputs?
    palette            = "mako",             # ChimeraX-compatible viridis palette
    start_frame        = 0,                  # skip first N frames
    end_frame          = None,               # None = process to final frame
    make_plot          = True,               # generate PNG heatmap
    analysis_type      = "protein",          # "protein" or "nucleic" (nucleic is
        still experimental)
    summary_n          = 3,                  # annotate top 3 regions
    manual_length_ns   = None,               # override time -> ns conversion
    log_transform      = True                # log-scale the RMSX values
)

# After completion, check ‘output_dir‘ for:
#   - chain_rmsx.csv       Columns: ResidueID, ChainID, slice_1.dcd, slice_2.dcd ...
#   - heatmap.png          full-heatmap visualization
#   - snapshots/*.pdb      PDBs with RMSX written to B-factor column
```

Supplemental Code Snippet 2: Example invocation of `run_rmsx_flipbook()`

```
from rmsx import run_rmsx_flipbook
# File paths for flipbook generation
pdb_file_flipbook   = "/path/to/structure.pdb"
dcd_file_flipbook   = "/path/to/trajectory.dcd"
output_dir_flipbook = "/path/to/results/rmsx_flipbook_run"
# Run RMSX Flipbook
run_rmsx_flipbook(
    topology_file          = pdb_file_flipbook,          # PDB/PSF input
    trajectory_file        = dcd_file_flipbook,          # DCD/XTC input
    output_dir             = output_dir_flipbook,        # where to save outputs
    num_slices             = 24,                         # split trajectory into 24
        windows
    slice_size             = None,                       # or fixed frames/window
    rscript_executable     = "Rscript",                  # path to Rscript executable
    verbose                = True,                       # print detailed logs (False
        to suppress)
    interpolate            = False,                      # smooth between slices (True
        to enable)
    triple                 = True,                       # include RMSD/RMSF flank
        plots
    overwrite              = True,                       # overwrite existing output
        directory
    palette                = "mako",                     # ChimeraX-compatible viridis
        palette
    spacingFactor          = "0.7",                      # spacing factor for flipbook
    start_frame            = 0,                          # starting frame index for
        analysis
    end_frame              = 1200,                       # ending frame index (None =
        final frame)
    flipbook_min_bfactor   = 0,                          # min B-factor value for
        coloring flipbook
    flipbook_max_bfactor   = 2.27,                       # max B-factor value for
        coloring flipbook
    log_transform          = False,                      # apply log scale to RMSX
        values (True/False)
    extra_commands         = [                           # additional custom ChimeraX
        commands
        'color /b fire brick',                           # example - color chain B red
        'cartoon byattr bfactor /a min:0.2 max:2.0',     # example - color by chain A
            min/max values
        'cartoon byattribute bfactor /b min:1 max:1'     # example - reset worm size of
             chain B
    ]
)
```

Supplemental Figures

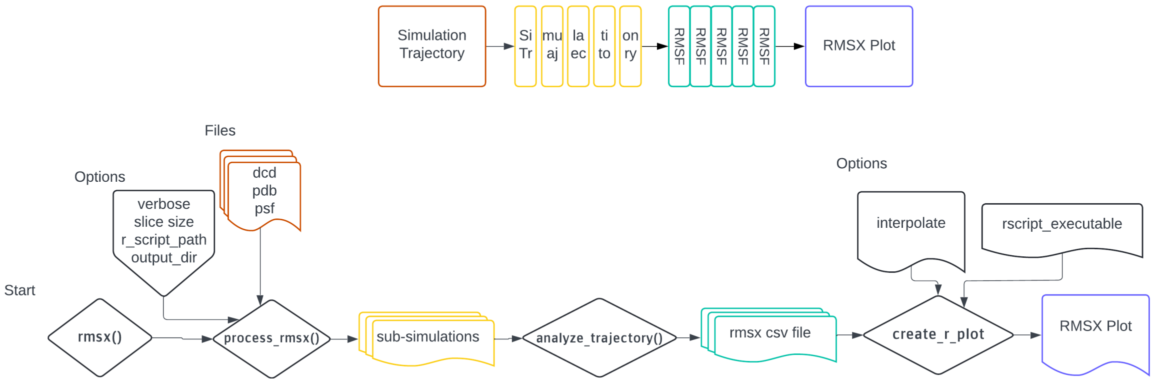

Supplemental Figure 1: Fig. S1. **RMSX Workflow Diagram.** Schematic representation of the RMSX pipeline, highlighting the key stages from raw molecular dynamics (MD) trajectory files to the generation of time-resolved fluctuation plots. The workflow begins with the input of simulation files (PSF, PDB, and DCD), along with user-defined options such as slice size and output directory. The `process_rmsx()` function segments the trajectory into consecutive sub-simulations, which are then analyzed via `analyze_trajectory()` to compute per-slice RMSF values. These are compiled into CSV files used for downstream visualization. The `create_r_plot()` function utilizes an R script to generate the final RMSX plot. Optional interpolation enhances the continuity of fluctuation patterns. The final output is a heatmap that captures both when and where residue-level fluctuations occur along the trajectory, facilitating high-resolution spatiotemporal analysis of protein motion.

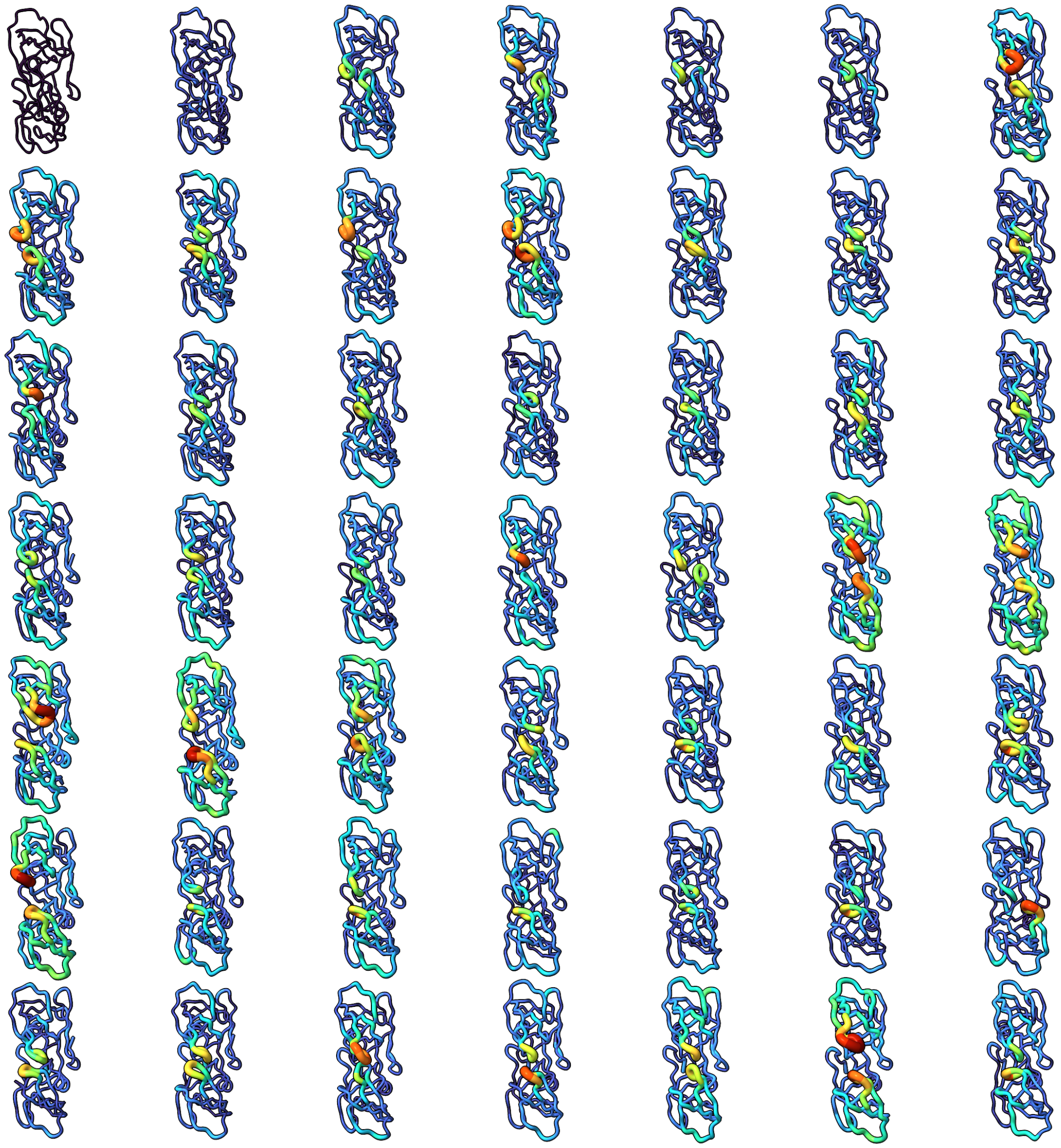

Supplemental Figure 2: **Shift-Map Flipbook for Protease (7×7 Grid)**. Sequential per-slice structural snapshots of the HIV-1 protease, colored and scaled by per-residue displacements relative to the first frame of the trajectory. Each panel in the 7×7 grid corresponds to a distinct time slice along the simulation, illustrating cumulative residue-level shifts from the initial reference conformation. Warmer colors and thicker tubes indicate larger deviations. This visualization highlights that emergence of alternative conformations over time that may not be apparent when analyzing shifts with respect to a single frame, thus reinforcing the importance of time-resolved analyses such as RMSX.

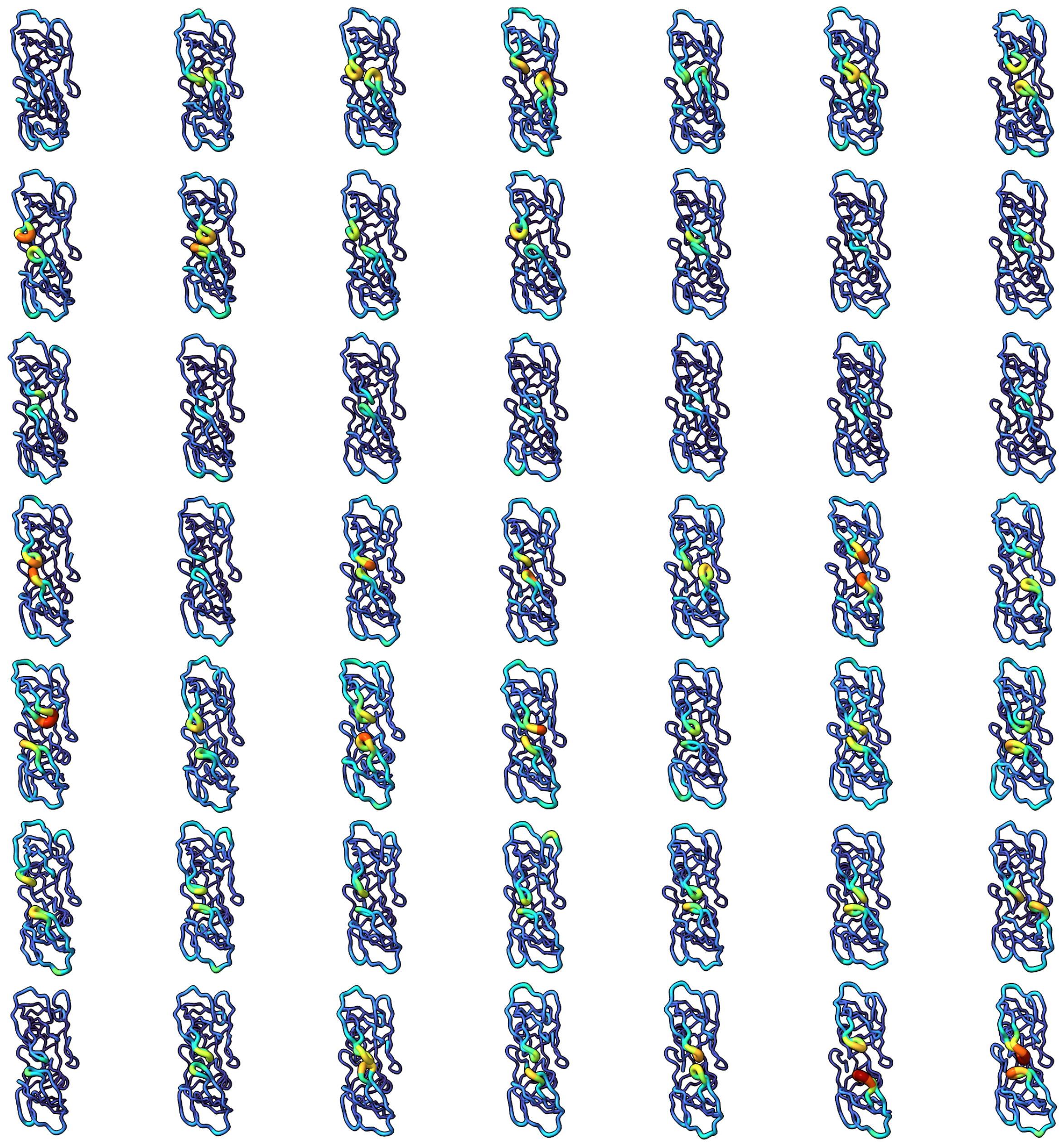

Supplemental Figure 3: Fig. S3. **RMSX Flipbook for Protease (7×7 Grid).** Time-resolved visualization of residue-level fluctuations in HIV-1 protease using RMSX values mapped onto each trajectory slice. As in Supp. Fig. 2, each panel represents a distinct window of simulation time, but here fluctuations are calculated within each slice, rather than relative to a fixed reference frame. Color and tube thickness reflect local positional variability, revealing transient dynamics and stable segments. In contrast to the shift-map approach, RMSX highlights fluctuations that are temporally confined and can return to baseline. Together, these two visualizations provide complementary views: shift maps detect accumulated drift, while RMSX pinpoints when transient conformational changes occur.

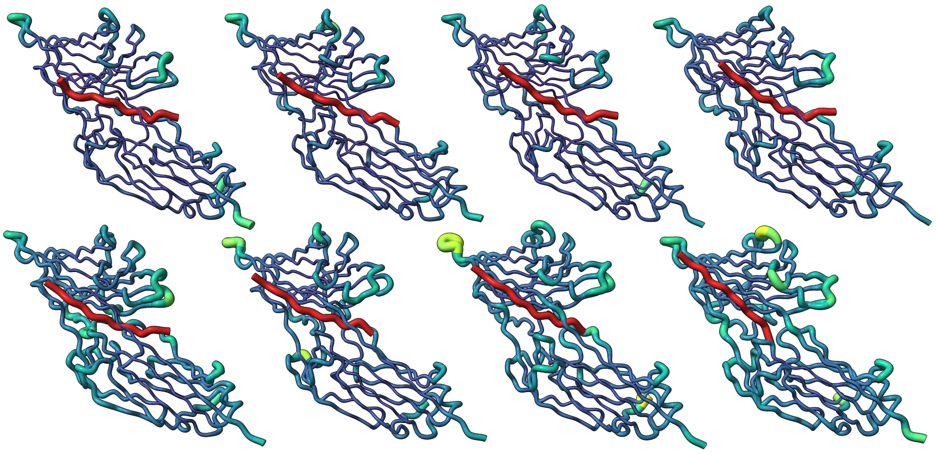

Supplemental Figure 4: **Time-resolved RMSX Flipbook of SdrG (2×4 Grid)** Snapshots of the SdrG–fibrinogen complex rendered with RMSX values mapped to the B-factor field, visualized using ChimeraX with tube color and thickness representing local residue fluctuations. Each panel corresponds to a consecutive slice of the trajectory, using the same time windows as described in the main text. This series highlights dynamic shifts in the N2 and N3 loop regions, which undergo progressive structural rearrangements under applied force. The visualization captures transient fluctuations that reflect the stepwise unfastening central to the "dock, lock, and latch" mechanism, enabling direct observation of when and where key conformational changes occur during dissociation, in loop.

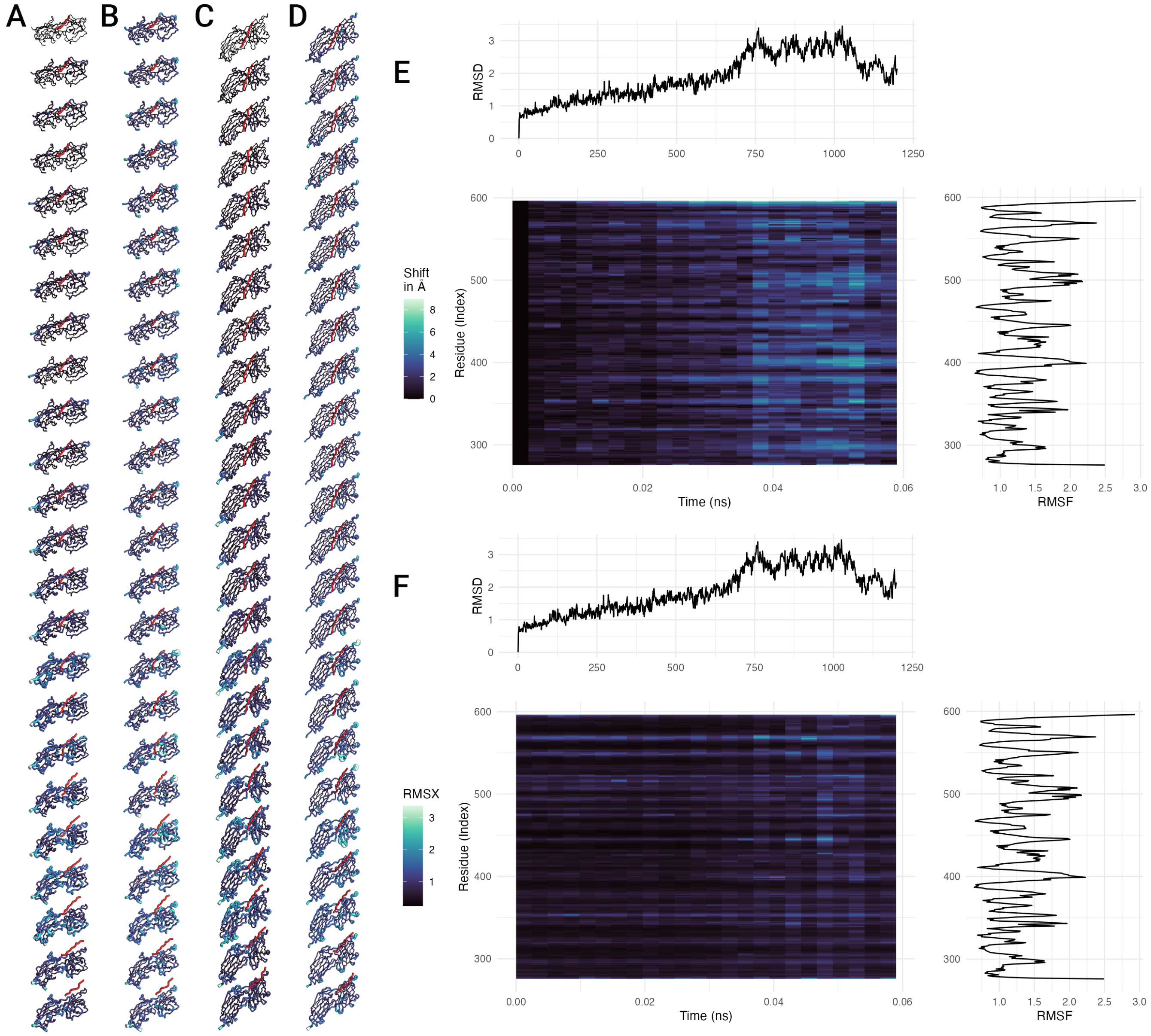

Supplemental Figure 5: **Comparison of RMSX and Trajectory Map. A.** Time-ordered structural snapshots of the SdrG complex showing per-residue displacement (Shift Map) relative to the initial frame, rendered from the side. Residues are colored and scaled by the magnitude of their cumulative positional shift, revealing progressive conformational drift. **B.** Corresponding RMSX flipbook from the same side view, illustrating per-slice fluctuations computed independently within each trajectory segment. This highlights transient residue motions that may not lead to permanent displacement. **C.** Shift Map snapshots shown in the main-text orientation, emphasizing cumulative loop drift from the perspective used in primary figures. **D.** RMSX snapshots in the same main-text orientation, illustrating how time-localized flexibility appears across the trajectory, even in regions that ultimately return to their original positions. **E.** Quantitative comparison of Shift Map metrics: the top panel shows the global RMSD trajectory of the complex over time; the middle heatmap displays per-residue displacement across simulation time; and the right plot summarizes average per-residue fluctuation (RMSF) derived from shift values. **F.** Equivalent plots based on RMSX: the same RMSD trajectory is shown as a RMSX heatmap, and an RMSF profile derived from slice-wise fluctuation values (right). This comparison illustrates how shift maps are sensitive to net positional drift, while RMSX more effectively captures localized, time-dependent flexibility.

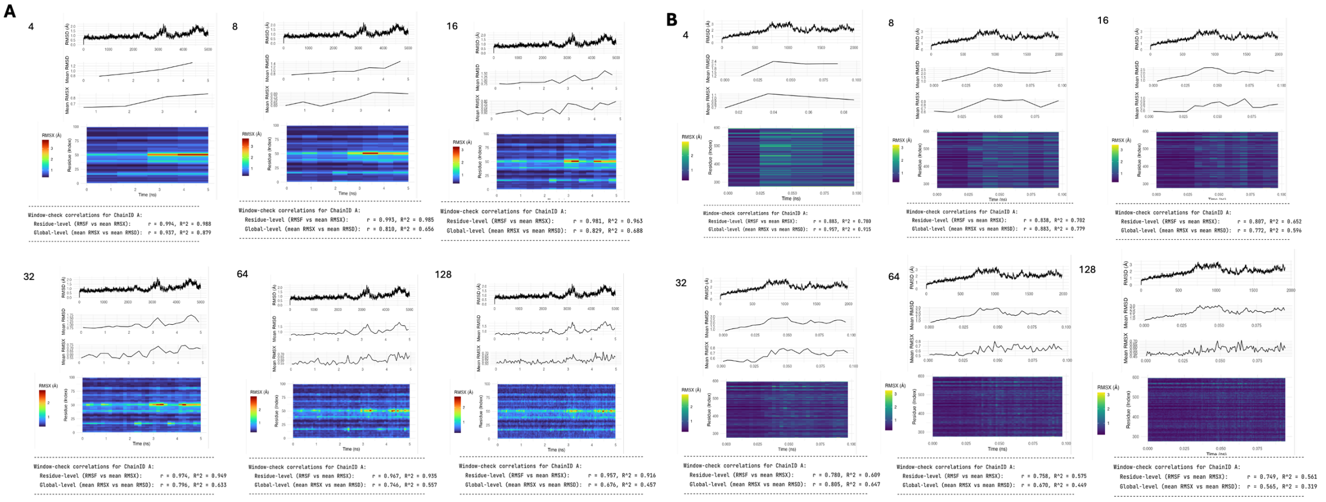

Supplemental Figure 6: **Window-Size Comparison in Protease and SdrG1. A.** RMSX flipbooks for Protease chain A generated using window sizes spanning 4–128 slices (corresponding to approximately 1,250–78 frames per window). The `window_check` option provides synchronized views of mean RMSX and mean RMSD, allowing the user to visually evaluate their agreement. The tool additionally reports the coefficient of determination (R^2) between mean RMSX and mean RMSD, along with comparisons to RMSF, to assist in selecting an appropriate window size.

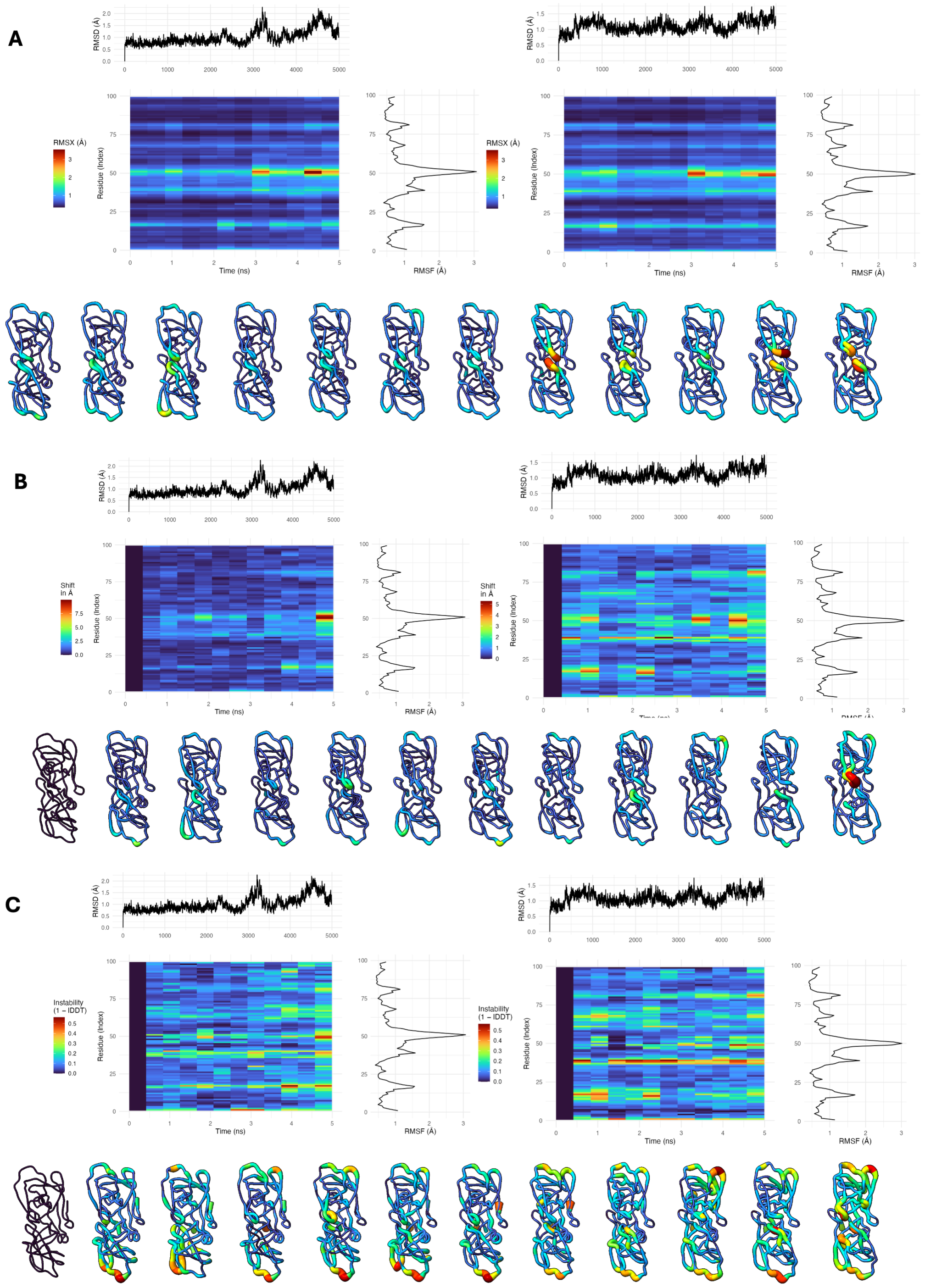

Supplemental Figure 7: **Protease RMSX, Trajectory Map, and lDDT Comparison. A.** RMSX flipbook of the protease used in Figures 2 and 3, computed using a 412-frame window and divided into 12 trajectory slices. Residues are colored and scaled by slice-wise fluctuation, highlighting pronounced motion around the active site. **B.** Corresponding trajectory-map flipbook (no sliding window), showing cumulative per-residue displacement over time. Because the trajectory map depends on instantaneous snapshot alignment, mobile opening of the active site may be missed when not captured in a displayed frame. **C.** Inverted lDDT flipbook (1–lDDT) generated from 300 snapshots using a standard 15 Å cutoff and a 2–4–8 neighborhood. The inverted lDDT pattern emphasizes peripheral flexibility rather than motion centered on the active site.
